# Construction of a high-density genetic map and detection of a major QTL of resistance to powdery mildew (*Erysiphe necator* Sch.) in Caucasian grapes (*Vitis vinifera* L.)

**DOI:** 10.1186/s12870-021-03174-4

**Published:** 2021-11-11

**Authors:** Tyrone Possamai, Sabine Wiedemann-Merdinoglu, Didier Merdinoglu, Daniele Migliaro, Gloria De Mori, Guido Cipriani, Riccardo Velasco, Raffaele Testolin

**Affiliations:** 1 Department of Agricultural, Food, Environmental and Animal Sciences, University of Udine, via delle Scienze 206, 33100 Udine, Italy; 2 CREA - Research Centre for Viticulture and Enology, viale XXVIII Aprile 26, 31015 Conegliano, TV Italy; 3 INRAE, Université de Strasbourg, SVQV UMR-A 1131, 28 rue de Herrlisheim, 68000 Colmar, France; 4 Institute of Applied Genomics, Science & Technology Park “Luigi Danieli”, via Jacopo Linussio 51, 33100 Udine, Italy

**Keywords:** Resistance genes, *Ren* loci, Grape breeding, Powdery mildew phenotyping

## Abstract

**Background:**

*Vitis vinifera* L. is the most cultivated grapevine species worldwide. *Erysiphe necator* Sch., the causal agent of grape powdery mildew, is one of the main pathogens affecting viticulture. *V. vinifera* has little or no genetic resistances against *E. necator* and the grape industry is highly dependent on agrochemicals. Some Caucasian *V. vinifera* accessions have been reported to be resistant to *E. necator* and to have no genetic relationships to known sources of resistance to powdery mildew. The main purpose of this work was the study and mapping of the resistance to *E. necator* in the Caucasian grapes ‘Shavtsitska’ and ‘Tskhvedianis tetra’.

**Results:**

The Caucasian varieties ‘Shavtsitska’ and ‘Tskhvedianis tetra’ showed a strong partial resistance to *E. necator* which segregated in two cross populations: the resistant genotypes delayed and limited the pathogen mycelium growth, sporulation intensity and number of conidia generated. A total of 184 seedlings of ‘Shavtsitska’ x ‘Glera’ population were genotyped through the Genotyping by Sequencing (GBS) technology and two high-density linkage maps were developed for the cross parents. The QTL analysis revealed a major resistance locus, explaining up to 80.15% of the phenotypic variance, on ‘Shavtsitska’ linkage group 13, which was associated with a reduced pathogen infection as well as an enhanced plant necrotic response. The genotyping of 105 Caucasian accessions with SSR markers flanking the QTL revealed that the resistant haplotype of ‘Shavtsitska’ was shared by ‘Tskhvedianis tetra’ and a total of 25 Caucasian grape varieties, suggesting a widespread presence of this resistance in the surveyed germplasm. The uncovered QTL was mapped in the region where the *Ren1* locus of resistance to *E. necator*, identified in the *V. vinifera* ‘Kishmish vatkana’ and related grapes of Central Asia, is located. The genetic analysis conducted revealed that the Caucasian grapes in this study exhibit a resistant haplotype different from that of Central Asian grape accessions.

**Conclusions:**

The QTL isolated in ‘Shavtsitska’ and present in the Caucasian *V. vinifera* varieties could be a new candidate gene of resistance to *E. necator* to use in breeding programmes. It co-localizes with the *Ren1* locus but shows a different haplotype from that of grapevines of Central Asia. We therefore consider that the Caucasian resistance locus, named *Ren1.2*, contains a member of a cluster of R-genes, of which the region is rich, and to be linked with, or possibly allelic, to *Ren1*.

**Supplementary Information:**

The online version contains supplementary material available at 10.1186/s12870-021-03174-4.

## Background

The introduction into Europe of the grape mildews (*Plasmopara viticola* Berl. & De Toni and *Erysiphe necator* Sch.) in the middle of the nineteenth century caught the viticulture of the ancient world unprepared to cope with those diseases. Devastation occurred because the European grape, *Vitis vinifera* L., did not carry any defence mechanism to contrast the diseases, while American grapes, being coevolved with the pathogens, had time to develop appropriate defence mechanisms [1].

The investigation of American and Asian native grapes, initiated soon after the grape mildews spread [2], led to the identification of numerous sources of resistance [3–6], which were systematized with the improvement of genetic analyses and the more recent development of molecular tools. Within just a few decades such work led to the identification of 32 QTL of resistance to downy mildew and 14 QTL of resistance to powdery mildew [e.g. [7–9]; www.vivc.de]. Many of these loci appear as minor QTL as they explain little phenotypic variance (in some cases less than 10%). However, the number of major QTL available is reasonably sufficient to commit grape breeders to combine these resistance loci together and introgress them into the *V. vinifera* genetic background. For downy mildew, the loci that breeders work with are *Rpv1* [10, 11], *Rpv3* [12, 13], *Rpv10* [14] and *Rpv12* [15] that explain large phenotypic variability and for which in most cases gene sequences and/or tightly associated markers have been identified. The major QTL of resistance to powdery mildew discovered up to now and exploited in grape breeding are *Run1* [16, 17], *Ren1* [18, 19], *Ren3* [20], *Ren4* [21] and *Ren9* [22, 23]*.*

Almost all these loci are originated in wild American and Asian species, but there are a few exceptions. Occasionally, resistance loci have been found in *V. vinifera* accessions of Central Asia, where the limited use of sprays against pathogens, the maintenance of chance seedlings originated in the vineyards and the presence of wild seedlings of *V. vinifera* subsp. *sylvestris* allowed European grapevines to develop or introgress limited but interesting sources of resistance. The first case of resistance reported in *V. vinifera* is the *Ren1* locus, which confers resistance to *E. necator* and was found in ‘Kishmish vatkana’, a table grape variety cultivated in Uzbekistan [18]. A deeper analysis of Central Asian germplasm revealed that other cultivated and wild grape accessions from Uzbekistan and neighbouring countries (Tajikistan, Turkmenistan and Afghanistan), some of which have ‘Kishmish vatkana’ kinship, carried the same QTL [19, 24]. Hence, the exploration of Caucasian germplasm uncovered further sources of resistance to downy mildew [8, 25, 26]. Interestingly, some of these accessions deploy unique resistance patterns, which include the overexpression of genes related to pathogen recognition, the synthesis of antimicrobial compounds, and structural barriers [27].

With the aim of mining new sources of resistance in the large reservoir of grape germplasm disseminated in the area from the Caucasian mountains to Central Asia, we screened 105 Caucasian accessions conserved at the CREA - Research Center for Viticulture and Enology (CREA-VE) germplasm collection, performed targeted controlled crosses, and mapped a QTL in a region of the linkage group 13 that is rich in genes associated with plant defence responses [19].

## Results

### Evaluation of the resistance to *E. necator* in the two cross populations

Segregation of the resistance to *E. necator* was evaluated in two populations produced by crossing the Caucasian varieties ‘Shavtsitska’ and ‘Tskhvedianis tetra’, both partially resistant to the pathogen, with the susceptible grapevine varieties ‘Glera’ and ‘Chardonnay’, respectively.

The resistance was evaluated by leaf disc bioassays and phenotyping was performed for a total of 264 seedlings of 50042 - ‘Shavtsitska’ x ‘Glera’ population (158 were evaluated three times and 106 twice) and 67 seedlings of 50041 - ‘Chardonnay’ x ‘Tskhvedianis tetra’ population (58 were evaluated twice and 9 once). Only seedlings displaying an optimal health state were sampled in each experiment. The phenotypic data for the offspring and parents were recorded daily between 2–3 to 10–11 days post-infection (dpi). The variables observed were *E. necator* mycelium growth and sporulation intensity, the mean number of conidia per conidiophore, produced conidia number per disc at 10 dpi, the calculated relative Area Under Disease Pressure Curve (rAUDPC) indexes and plant necrosis production.

**Fig. 1:**
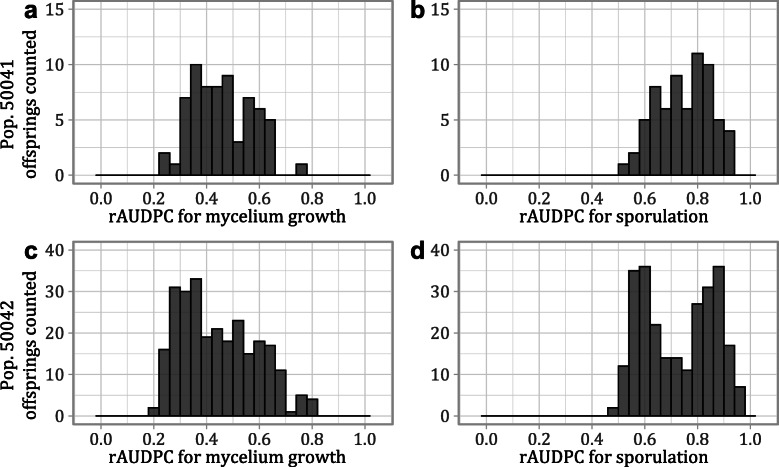
Calculated rAUDPC data for the offsprings of the cross populations. The plots show the frequency of the rAUDPC indexes calculated for the offspring (averaged data from two or three experiment replicates) of the cross populations 50041 (plot **a**-**b**) and 50042 (plot **c**-**d**) in the evaluation of the resistance to *E*. necator

Details on the experiments and descriptive statistics for some data related to *E. necator* infection are shown in the Additional file 1: Table S1. The offspring of populations 50041 and 50042 showed a wide variability of resistance phenotypes, hence the segregation of trait (Fig. 1). *E. necator* mycelium growth and sporulation intensity provided the most reliable results to evaluate the progress of the disease. In the Additional file 2: Fig. S1. the distributions of such data in different dpi and experiments are displayed. The individuals showed either susceptible-like phenotypes (similar to ‘Glera’, ‘Chardonnay’ and to the susceptible *V. vinifera* variety control ‘Cabernet sauvignon’) with a rapid progression of the infection, or a partial resistance to *E. necator* (similarly to the parental plants ‘Shavtsitska’ and ‘Tskhvedianis tetra’) with a delayed and more limited pathogen growth and sporulation. The differences between resistant and susceptible seedlings were greater at 5 and 7 dpi, compared to previous and later dpi, and were summarized by the rAUDPC indexes. According to the observed differences, depending on the variable, dpi, experiment and population considered phenotypes were differently distributed and likely bimodal distributions were also observed (Fig. 1; Additional file 2: Fig. S1). Data of 50042 population appeared to be more often bimodal than data from population 50041 probably because for such population more seedlings, experiment replicates and consistent results were obtained. Finally, the offspring classification following the rAUDPC scores of the cross parents suggested that the resistance in both populations segregated in a Mendelian way with a ratio of 1:1 (Table 1).

**Table 1:** Segregation of the resistant trait in the cross populations. Offspring were classified susceptible or resistant on the basis of their rAUDPC indexes for the pathogen mycelium growth and sporulation (averaged data from two or three experiment replicates) compared to the calculated values for the control genotypes and cross parents

Population	Phenotypic data	Susceptibility interval^a^	Resistance interval^b^	Susceptible offsprings	Resistant offsprings	***p***-value χ2 test (1:1)
50041	rAUDPC for mycelium growth	0,15-0,44^c^	0,42-0,70	32	35	0,71
50042			0,65-0,91	141	123	0,27
50041	rAUDPC for sporulation intensity	0,45-0,72^c^	0,87-0,94	40	27	0,11
50042			0,93-1,00	132	132	1,00

^a^determined as 5th and 95th percentile of the rAUDPC values calculated for the susceptible control genotypes and cross parents

^b^for population 50041 determined as the minimum and the maximum rAUDPC value calculated for the resistant cross parent; for population 50042 determined as 5th and 95th percentile of the values calculated for the resistant cross parents

^c^thresholds values distinguishing susceptible and resistant offsprings

The offspring of the two populations displayed a similar global rate of infection to one another and between experiment replicates (Fig. 1; Additional file 1: Table S1; Additional file 2: Fig. S1). The data of the variables recorded were often correlated with each other (Additional file 3: Fig. S2) and high correlations between experiment replicates were also noted. For instance the rAUDPC for pathogen sporulation intensity (rAS) ranged between 0.42 (recorded for the experiments conducted on population 50041) and 0.69 (recorded between the 2nd and 3rd experiments conducted on population 50042) resulting the most reproducible variable (Additional file 3: Fig. S2).

### DNA sequencing and SNP calling

A total of 184 seedlings of population 50042 and the cross parents ‘Shavtsitska’ and ‘Glera’ were genotyped through a Genotyping by Sequencing (GBS) approach. The sequencing produced a total of 498 million reads with an average read pair count per sample of 2.5 million and a coefficient of variation of 36%. Reads were aligned to the ‘PN40024’ grape reference genome 12X.v2. The BAM records were analysed with Stacks that retained 596.5 million (62.9%) primary alignments and discarded 151.2 million (15.9%) alignments with insufficient mapping qualities and 200.7 million (21.2%) unmapped alignments; 40.1 to 67.7% records per sample were kept. The Stacks analysis identified 695,985 loci and an effective per-sample mean coverage of 22.1x (stdev = 7.2x, min = 5.1x and max = 44.0x). One SNP per locus was retained eliminating the variant sites with the lowest quality and a minimum allele frequency below 5%. Finally, 139,318 SNP variants were kept.

### Linkage maps of ‘Shavtsitska’ and ‘Glera’

A preliminary SNP analysis discarded 118,453 markers having a genotyping rate below 90% and 6181 showing not expected segregation patterns in the offspring and/or parents. The remaining 14,684 markers were divided into chromosomes (chr) according to their position in ‘PN40024’ and in two parental datasets to develop the linkage maps (pseudo-testcross mapping strategy): 6941 SNP segregated from ‘Shavtsitska’ and 7737 from ‘Glera’. About 61% of the markers per parent resulted co-segregating and were filtered retaining the SNP with low distorted segregations and missing data. The Minimum Spanning Tree map (MSTmap) algorithm confirmed the markers grouping in the 19 reference chromosomes and separated about 2% of SNP from the assigned linkage group (LG). After that, 11% of markers, with issues in the linkage maps (e.g. low mean association-LOD value/high recombination fraction), and one individual (seedling 7067z) showing more than 200 putative crossovers (an extraordinary number in comparison to the other seedlings) were manually filtered.

**Fig. 2:**
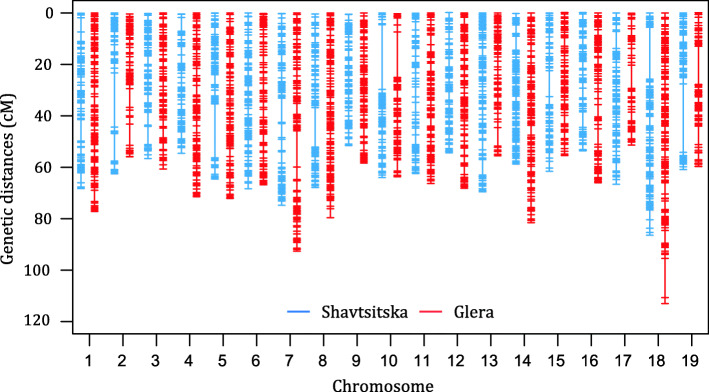
GBS-based genetic maps of ‘Shavtsitska’ and ‘Glera’. Chromosomes are numbered and oriented according to the grape ‘PN40024’ grape reference genome 12X.v2. ‘Shavtsitska’ (blue) and ‘Glera’ (red) maps are depicted by different colours

Thus, the final SNP datasets were composed of 183 individuals and 2291 markers for ‘Shavtsitska’ and 2627 markers for ‘Glera’. The maps were reanalysed by the MSTmap algorithm to improve the marker order and by the Lander-Green algorithm and Kosambi mapping function to define the genetic distances: ‘Shavtsitska’ and ‘Glera’ maps covered a total of 1205 cM and 1315 cM, respectively. Parental maps are shown in Fig. 2 and the number of markers and length of each linkage group are reported in Table 2. The Additional file 4: Fig. S3 shows the genetic maps with the SNP codes.

**Table 2:** Details for the parental genetic maps of ‘Shavtsitska’ and ‘Glera’

LG	Shavtsitska map			Glera map		
	Markers	LG length cM	Largest gap cM	Markers	LG length cM	Largest gap cM
**1**	131	68.2	8.4	159	77.2	4.4
**2**	72	62.5	21	98	55.5	8.3
**3**	116	56.4	7.1	139	60.6	3.8
**4**	112	54.2	5.3	166	71.4	3.8
**5**	113	64.5	20.9	169	72.2	3.2
**6**	125	68.4	2.4	131	66.6	3.3
**7**	157	74.6	9.4	146	92.6	14.1
**8**	110	67.5	15.3	191	79.6	3.5
**9**	104	51	2.8	115	58.4	3.3
**10**	119	64	25.2	102	63.5	17.7
**11**	108	62.4	3.9	122	66.3	3.9
**12**	138	54.6	3.3	138	68.2	3.3
**13**	166	69.5	6	112	55.5	7.2
**14**	138	58.5	5.5	216	81.6	3.9
**15**	100	61.5	10	107	55.6	3.3
**16**	88	53.8	6	122	66.1	6.1
**17**	131	66.6	6.6	79	51.2	9
**18**	166	86.4	20.2	198	113	15.3
**19**	97	60.9	26.6	117	59.8	14.9
**Total**	2291	1205.5	26.6	2627	1314.9	17.7

**Fig. 3:**
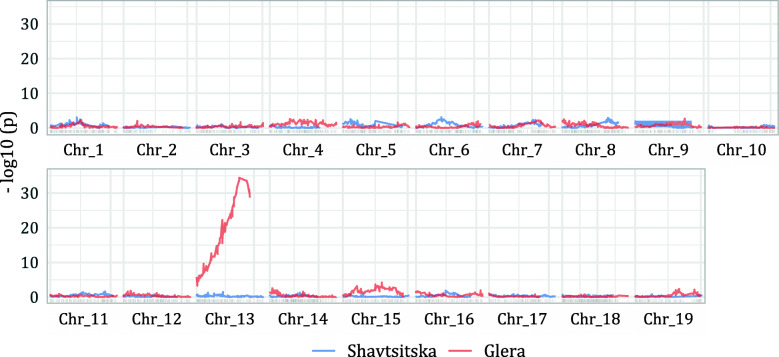
Markers segregation distortions along the genetic maps. On the y-axis the negative log10 of p-values in chi-square tests (−log10(p)) comparing segregation frequencies of the alleles of the SNP retained in the genetic maps of ‘Shavtsitska’ (blue) and ‘Glera’ (red). The higher values for ‘Glera’ chr 13 demonstrates the presence in the LG of significant segregation distortions from the expected Mendelian ratio of 0.5 (e.g. a -log10(p) value of 2 corresponding to a p-value of 0.01). Vertical grey breaks are positioned every 30 cM on the LG of the genetic maps

The SNP markers showed a complete coverage of the chromosomes of both maps. Marker density appeared higher for ‘Glera’ and the maximum distance between markers was usually below 5 cM. The ‘Shavtsitska’ map showed, in particular, five gaps of about 20 cM in the LG 2, 5, 10, 18 and 19 (Fig. 2; Table 2; Additional file 4: Figure S3; Additional file 5: Fig. S4). The observed gaps matched with genomic regions having lower densities of GBS markers (e.g. in chr 2 and 19) except the gaps in LG 10 of both maps which arisen despite the good coverage of the chromosome. Unexpectedly, most markers showed distorted segregations in ‘Glera’ LG 13. The segregations showed that one end of a chromatid of ‘Glera’ was less inherited in the offspring (Fig. 3). Provided that markers with distorted segregations did not alter the linkage order and distances, they were retained. Finally, the genetic maps showed a good correlation between the genetic order and physical position of SNP in ‘PN40024’ except for a few local inversions of small marker groups (e.g. in chr 3 and 5) (Additional file 5: Fig. S4).

### QTL analysis for the resistance to *E. necator*

**Fig. 4:**
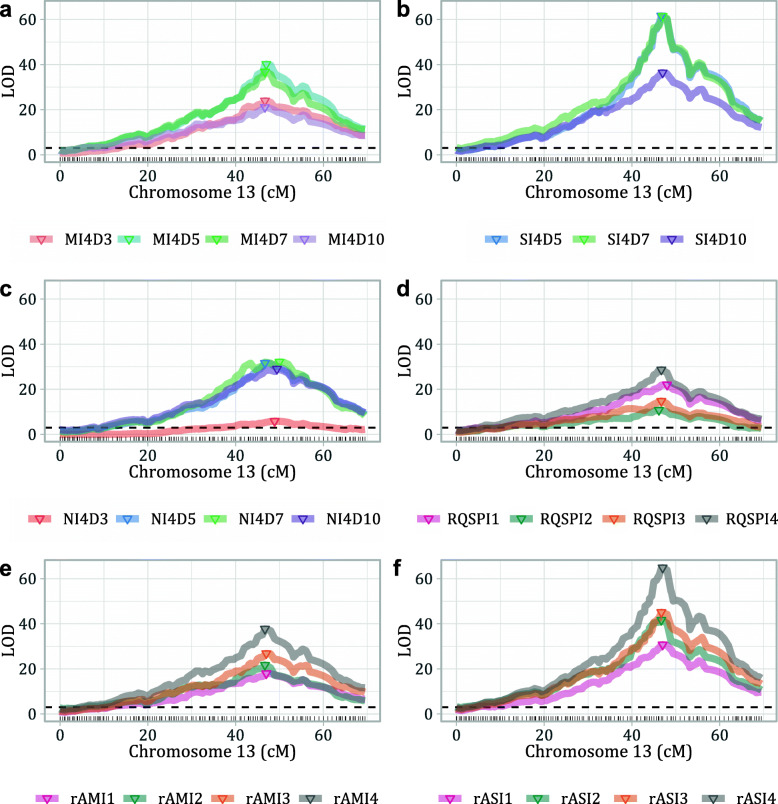
Interval mapping results for the QTL of resistance to *E. necator* identified in ‘Shavtsitska’ chr 13. Graphs a-b-c show the LOD values for the analysis carried out with the averaged data from 3 experiments (code I4) for pathogen mycelium growth (letter M-plot **a**), sporulation (S-plot **b**) and plant necrosis frequency (N-plot **c**) assessed at 3–5–7-10 dpi (D-different colours). Graphs **d**-**e**-**f** show the LOD values for the analysis carried out with conidia counts by Malassez chamber square-root transformed (RQSP-plot **d**), rAUDPC for pathogen mycelium growth (rAM-plot **e**) and sporulation (rAS-plot f) for the experiment replicates 1-2-3 (I-different colours) and the averaged data (I4-grey)

The mapping of the QTL of resistance to *E. necator* was performed by using the phenotypes of the 183 seedlings retained for the parental maps construction. The analysis identified a major QTL in ‘Shavtsitska’, and none in ‘Glera’. The interval mapping (IM) procedure located the QTL in the chr 13 of ‘Shavtsitska’ at about 47 cM from the top and in an interval of about 2.2 cM with most of the phenotypic data series processed (infection variables, different experiments and dpi of observation) (Fig. 4; Table 3; Additional file 6: Table S2). The calculated significance LOD-thresholds usually varied between 2.6 and 3.1. The LOD peak scores for the IM carried out with the averaged mycelium growth data varied from 23.78 to 40.17 according to the time of observation (dpi); for the mycelium growth at 5 dpi a maximum explained variance of 63.46% was recorded. The analysis with the sporulation intensity averaged values showed LOD peak scores between 36.41 and 61.45 and a maximum explained variance of 77.62% for observations made at 7 dpi. The plant necrotic response data had LOD peak values between 5.87 and 31.65 and a maximum explained variance of 54.91% for 5 dpi observations. The final count of conidia by Malassez chamber provided LOD peak values between 10.80 and 28.68 and a maximum explained variance of 50.68%. The rAUDPC indexes gave similar or better results in term of LOD peaks if compared with the single time-course observations: the maximum LOD peak values for the rAUDPC for mycelium growth and sporulation were 37.72 and 64.88, respectively, while their explained variance reached up to 61.30 and 80.15%, respectively (Fig. 4; Table 3; Additional file 6: Table S2).

**Table 3:** Proprieties of the significant QTL for the resistance to *E. necator* identified in ‘Shavtsitska’. List of the LOD-based largest QTL for the observed infection variables identified in the chromosome 13 of ‘Shavtsitska’ and information on their LOD scores, explained variance, map position, interval and associated SNP markers

Phenotypic data	Chr	LOD score	***p***-value	Expl. var. %	Pos. cM	Nearest marker	Bayes Conf. Intervals (lower and upper limits, α = 0.95)	
							Pos. cM	Markers
Mycelium growth 5 dpi	13	40.17	< 0.001	63.46	47.0	SNP_13_ 18,102,346	46.70; 48.89	SNP_13_17909186; SNP_13_18213673
Sporulation intensity 7 dpi	13	61.45	< 0.001	77.62	47.0	SNP_13_ 18,102,346	46.70; 48.89	SNP_13_18,102,346; SNP_13_18213673
Plant necrosis freq. 5 dpi	13	31.65	< 0.001	54.91	46.7	SNP_13_ 18,102,346	46.70; 48.89	SNP_13_17909186; SNP_13_18213673
Square root (n conidia/ml)	13	28.68	< 0.001	50.68	46.7	SNP_13_ 18,102,346	45.61; 48.89	SNP_13_15836674; SNP_13_18213673
rAUDPC for mycelium g.	13	37.72	< 0.001	61.31	46.7	SNP_13_ 18,102,346	46.70; 48.89	SNP_13_17909186; SNP_13_18213673
rAUDPC for sporulation i.	13	64.88	< 0.001	80.15	47.0	SNP_13_ 18,102,346	46.70; 48.89	SNP_13_17909186; SNP_13_18213673

The QTL analysis showed possible minor QTL in ‘Shavtsitska’ (e.g. in chr 14 for IM with sporulation intensity data) but the LOD values were just above the significance thresholds and QTL were not consistently detected in different experiments or with different phenotypic data (Additional file 6: Table S2). Finally, Kruskall-Wallis tests and multiple QTL analysis only confirmed the major QTL in ‘Shavtsitska’ chr 13 and did not evidence further QTL (data not shown).

**Fig. 5:**
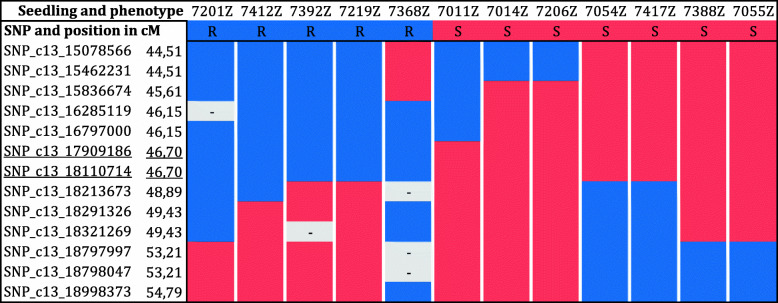
Recombinants for the QTL region associate to the resistance to *E. necator* in ‘Shavtsitska’. Markers are named with their physical position on the grape ‘PN40024’ grape reference genome 12X.v2. The susceptible (S) haplotype is in red and the resistant (R) haplotype is in blue. Missing data are in grey. Underlined the markers on ‘Shavtsitska’ map associate to the resistance QTL

Informative recombinants for the resistance locus identified in ‘Shavtsitska’ were searched among the genotyped progenies: five resistant and seven susceptible plants showed a recombination event between SNP_c13_15078566 and SNP_c13_18998373, that flank the region depicted in Fig. 5. Recombination events associated the locus involved in resistance to *E. necator* to a region of 1.4 Mb on the grape ‘PN40024’ grape reference genome between the SNP_c13_16797000 and the SNP_c13_18213673 (Fig. 5).

### Analysis of the ‘Chardonnay’ x ‘Tskhvedianis tetra’ population

SC8–0071-014 and Sc47_20 SSR markers, located in ‘PN40024’ chr 13 at 16.87 and 18.24 Mb, respectively, resulted tightly linked to the position of the resistance QTL identified in ‘Shavtsitska’. Both markers were assayed in a subsample of individuals of the breeding populations as well as in the resistant parents. The analysis revealed that the resistant progenies of both 50041 and 50042 populations has inherited the allele 149 of SC8–0071-014 and allele 208 of Sc47_20 markers.

**Fig. 6:**
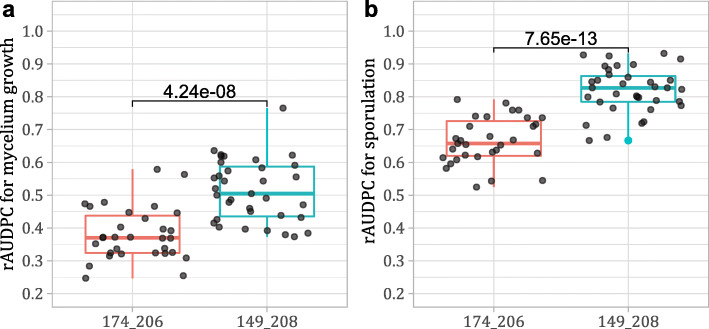
Resistance to *E. necator* segregation in the population 50041. The box-plots represent the averaged rAUDPC indexes for pathogen mycelium growth (plot **a**) and sporulation (plot **b**) of the offsprings of the cross ‘Chardonnay’ x ‘Tskhvedianis tetra’. Scores are grouped by their inherited haplotype: 174-206 (red) is the susceptible allele pair while 149-208 (blue) is the resistant haplotype. Above the box-plots the p-values from T-tests between the haplotype-derived groups

The screening with SC8–0071-014 and Sc47_20 markers was therefore extended to all 67 phenotyped offspring of 50041 population. A total of thirty-five individuals inherited the 149–208 haplotype from ‘Tskhvedianis tetra’, thirty-one the 174–206 haplotype and one individual was recombinant for the SSR. Highly significant phenotypic differences (*p*-value < 0.001 for T-test) for *E. necator* mycelium growth and sporulation intensity between haplotype-derived groups were found comparing the seedlings averaged rAUDPC values (Fig. 6). The haplotypes explained up to 55% of the phenotypic variance. Finally, the genetic and statistical analysis confirmed that the same resistance locus segregated in both cross populations and that it was shared from the Caucasian varieties ‘Shavtsitska’ and ‘Tskhvedianis tetra’.

### SSR genotyping of Caucasian grape germplasm

SC8–0071-014 and Sc47_20 markers were analysed in further 103 Caucasian varieties preserved at the CREA-VE grape germplasm repository. The haplotypic combinations at both SSR markers are summarized in Table 4 and reported in the Additional file 7: Table S3. For the marker SC8–0071-014 fifteen possible alleles were recorded; allele 149 (associated to the resistance in ‘Shavtsitska’) was found in twenty-five accessions. For Sc47–20 five alleles were detected and allele 208 (associated to the resistance in ‘Shavtsitska’) was counted thirty-nine times. The pair of alleles 149–208 was observed in twenty-five different varieties (Table 4; Additional file 7: Table S3).

**Table 4:** Screening with SSR of the Caucasian germplasm conserved at CREA-VE. Screening of 105 Caucasian *V. vinifera* accessions for their haplotypic combinations at the SSR markers SC8–0071-014 and Sc47_20 closely associated to the resistance to *E. necator*: the haplotype ‘147/- & 206/-’ is the resistance haplotype of ‘Kishmish vatkana’ (*Ren1*), while ‘149/- & 208/-’ the resistance haplotype of ‘Shavtsitska’. '-' any allele different from those in coupling with ‘Kishmish vatkana’ or 'Shavtsitska' resistance QTL

SC8–0071-014 (↓) and Sc47_20 alleles (→)	206/−	208/−	206/208	−/−	Total
**147/−**	2	0	0	6	8
**149/−**	0	19	4	1	24
**147/149**	0	1	0	0	1
**−/−**	8	14	1	50	73
**Total**	10	34	5	59	105

SC8–0071-014 and Sc47_20 markers were also associate to the *Ren1* locus. Analysis of ‘Kishmish vatkana’ and ‘Dzhandzhal kara’, the two grape cultivars of Central Asia where *Ren1* was identified, showed the resistant haplotype being 147–206. Such an allelic combination was found in only two Caucasian accessions. (Table 4; Additional file 7: Table S3).

### Characterization of the resistance in ‘Shavtsitska’ and ‘Tskhvedianis tetra’

The phenotypic effects of the resistance of the Caucasian accessions ‘Shavtsitska’ and ‘Tskhvedianis tetra’ and their progenies were studied by evaluating the *E. necator* conidia germination, hyphae and mycelium growth and conidiophores and conidia production (the pathogen life cycle) and the plant necrotic reactions from 1 to 11 dpi.

**Fig. 7:**
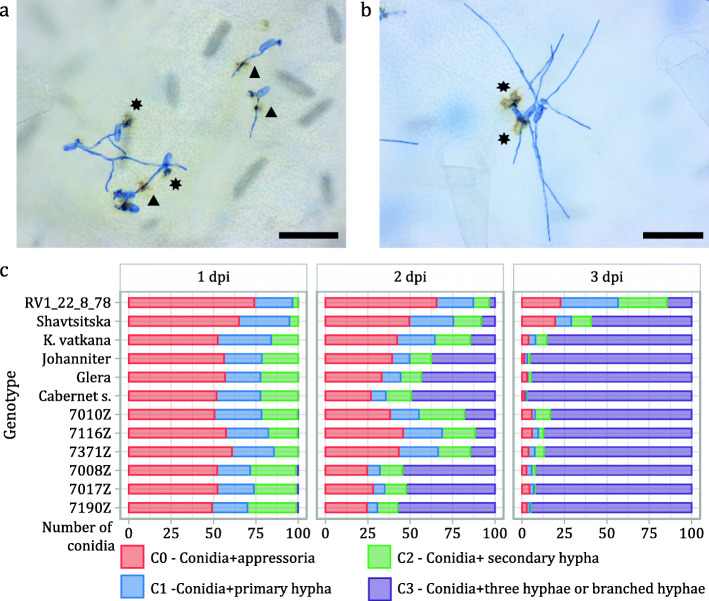
Summary of the conidia classification in Trypan-Blue bioassays. The images show the E. necator conidia development at 2 dpi on ‘Shavtsitska’ (**a**) and ‘Glera’ (**b**). In ‘Shavtsitska”, the pathogen growth was delayed and more limited than in ‘Glera’, furthermore plant necrotic response-HR (brown area) was present under the conidia appressoria (⎈) and under hyphae appressoria (▲) (magnification 200x and scale bar 100 μm). Below the conidia classification at 1-2-3 dpi following the Trypan-Blue staining (**c**). Class 0 (C0 - red) identifies conidia showing the appressoria, class 1 (C1 - blue) the conidia with the primary hypha, class 2 (C2 - green) the conidia with the secondary hypha and class 3 (C3 - violet) the conidia with the tertiary hypha and/or hyphae ramification. Seedlings ‘7010Z’, ‘7116Z’ and ‘7371Z’ are resistant offspring while ‘7008Z’, ‘7017Z’ and ‘7190Z’ are susceptible ones

The early response to *E. necator* of ‘Shavtsitska’ and its progeny was investigated between 1 and 3 dpi by microscope after Trypan-Blue staining and by scanning electron microscope (SEM). With the Trypan-Blue staining (Fig. 7), we observed 100 germinated conidia (germination rate of 97–99% on all the leaf discs) and we recorded that *E. necator* growth was delayed in resistant genotypes: on ‘Shavtsitska’, the conidia hyphae proliferation was already affected at 1 dpi, at this time point only 5% of conidia produced the secondary hypha on the host (symptom of an established successful interaction); on the resistant offspring and ‘Kishmish vatkana’, the delayed pathogen growth was recorded from 2 dpi when only 44% of the conidia had established a successful interaction. On the susceptible plants and on the control ‘Johanniter’, *E. necator* development was faster and about 26 and 64% of conidia developed two hyphae at 1 and 2 dpi, respectively. However, on all the resistant genotypes, except the control ‘RV1–22–8-78’, pathogen growth was not halted and at 3 dpi most of the conidia showed an established successful interaction and many hyphae (Fig. 7c; Additional file 8: Table S4). Finally, in Trypan-Blue staining experiments, resistant plants showed a frequent necrotic-hypersensitive response (HR) starting from 2 dpi that was recorded beneath the appressoria of both conidia and hyphae. On susceptible plants few necrosis were seen only close to the conidia appressoria (Fig. 7a-b).

**Fig. 8:**
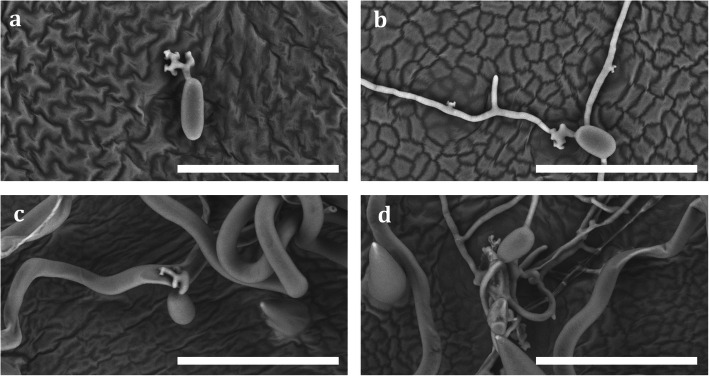
Insights recorded by scanning electron microscope (SEM). The images a-b show conidia with multilobed appressoria on resistant offspring (**a**) and conidia with less lobed appressoria on susceptible plants (**b**). The images c-d show for ‘Shavtsitska’ conidia fallen on prostrate hairs that do not successfully colonize the host (**c**) and conidia fallen on straight hairs that develop two or more hyphae (**d**) (magnification 1000x and scale bar 100 um)

Observations made at SEM supported the Trypan-Blue staining results and confirmed the different time course of hyphae proliferation on the resistant and susceptible genotypes. Furthermore, SEM revealed on resistant genotypes and resistant control plants frequent conidia with larger multilobed appressoria and hyphae that developed multiple new appressoria early. Finally, a lack of growth capability for conidia fallen on prostrate hairs of ‘Shavtsitska’ leaves was also observed; in such circumstances conidia collapsed if they did not produce an appressoria either on the leaf surface or on the straight hairs of the leaf (Fig. 8).

The stereomicroscope observations between 2 and 11 dpi still showed different rates of pathogen development on the studied plants. On ‘Shavtsitska’, ‘Tskhvedianis tetra’ and resistant offspring, at 2–3 dpi the pathogen developed only a few hyphae (score 7), while mycelium patches (score 5) were frequently observed on susceptible plants. The differences in the pathogen mycelium growth between genotypes increased at 4–5 dpi, persisted at 7 dpi and often even after: intermediate (score 7 and 5) and high (score 3 and 1) rates of infection were observed in resistant and susceptible plants, respectively. The resistance genotypes also affected many components of the *E. necator* sporulation: the latent period (emergence of conidiophores) was delayed up to 7 dpi, conidiophores spread and density was limited (score of 7 and 5) and conidia production was reduced (at 10–11 dpi between 1 and 3 conidia per conidiophore were recorded). On susceptible plants, incipient conidiophores were observed since 4 dpi, sporulation covered all the leaf discs (score 3 and 1), at 10–11 dpi up to 6 six conidia per conidiophore were recorded and, on average, 2–3 times more conidia per disc were counted by Malassez chamber. Finally, as confirmed by the QTL analysis, necrosis frequency was higher in resistant plants and clearly notable since 5 dpi. On susceptible genotypes, the plant hypersensitive response was weak, infrequent and limited to the beginning of the infection. The Additional file 8: Table S4 provides the phenotypic scores per offspring and cross parent recorded during the *E. necator* infections. The Additional file 9: Fig. S5 displays representative ‘resistant’ and ‘susceptible’ phenotypes of two individuals of cross population 50042 at the stereomicroscope observations.

In conclusion, the effects of ‘Shavtsitska’ and ‘Tskhvedianis tetra’ resistance seems to start before 1 dpi and extend to all the pathogen life cycle affecting hyphae proliferation, mycelium growth, latent period and sporulation amount.

## Discussion

Several Caucasian *V. vinifera* were recently described to be resistant to *E. necator* [28]. A preliminary survey did not identify a genetic relationship of these accessions to known sources of resistance (data not shown) and suggested that unexplored resistance determinants could be present in the Caucasian germplasm. With the aim of investigating such a hypothesis, the Caucasian resistant varieties ‘Shavtsitska’ and ‘Tskhvedianis tetra’ were crossed with the susceptible varieties ‘Glera’ and ‘Chardonnay’. Phenotyping bioassays were performed on the cross parents and offspring. The two Caucasian varieties showed a partial resistance to *E. necator* that segregates in the progeny and resulted as being controlled by a major resistance QTL located in chromosome 13.

### Caucasian grape varieties show a resistance to *E. necator*

The Caucasian grapevines showed both similar and different resistance phenotypes in response to *E. necator* in comparison with the varieties carrying known resistance loci.

‘Shavtsitska’ and its offspring contrasted the pathogen hyphal growth early, which at 1–2 dpi resulted strongly delayed compared to the susceptible control ‘Cabernet sauvignon’ and the susceptible cross parent ‘Glera’ (Fig. 7; Additional file 8: Table S4). Previous studies reported substantial differences at 2 and 3 dpi for *E. necator* development on *Vitis* accessions carrying different resistance sources [29–31]. *Run1* locus was described to halt the *E. necator* conidia penetration and hyphae elongation early at 1–2 dpi, reacting at the infection sites through a programmed cell death (PCD) deployment, callose depositions and reactive-oxygen-species (ROS) production [29, 30]. ‘Kishmish vatkana’ and other varieties carrying *Ren1* were described to delay powdery mildew infection [18], activating the plants reactions with lower intensity and later in comparison to *Run1*-mediated resistance [30]. Zendler et al. [22] observed that *Ren3*/*Ren9* effects on *E. necator* development were evident from 5 dpi. Our results, for the control genotypes ‘RV1–22–8-78’ (carrying the *Run1*), ‘Kishmish vatkana’ (carrying the *Ren1*) and ‘Johanniter’ (carrying the *Ren3* and *Ren9* loci) agreed with previous studies and allowed it to be speculated that ‘Shavtsitska’ had an effective ‘post-penetration reaction’ to *E. necator*: the variety did not halt the pathogen growth and showed the HR response, associable to a plant PCD [29] deployment, beneath the appressoria of both conidia and hyphae (Fig. 7). Preliminary studies suggested that PCD could be the key reaction to *E. necator* of ‘Shavtsitska’ because the callose depositions appeared very limited in the Caucasian variety, in comparison to the control varieties carrying the *Run1* or *Ren1* loci (data not shown).

According to SEM observations, *E. necator* produced larger multilobed appressoria from conidia and multiple new appressoria from hyphae on the studied resistant varieties (Fig. 8). Expanded and frequent appressoria in powdery mildew infections were already observed in resistant *Vitis* spp. accessions [32, 33]. Thus, such events confirmed that the pathogen on the studied resistant plants has more difficulties in establishing effective interactions due to the host response. SEM images also revealed that the conidia falling on prostrate airs of the leaves fail to develop mycelium (Fig. 8). Leaf hairs can influence pathogen infections, acting as a physical barrier or influencing the leaf micro-environmental conditions [34]. While a role of trichomes was often proposed in favouring grape resistance to *P. viticola* [3, 35, 36], no reference was found about their possible effects on the foliar resistance to *E. necator*. Our conclusions on this topic need to be confirmed because among all studied accessions only ‘Shavtsitska’ showed a high density of prostrate hairs.

‘Shavtsitska’, ‘Tskhvedianis tetra’ and their resistant progenies showed a partial resistance to *E. necator*. It means that the pathogen was able to complete his lifecycle but its development was contrasted by the host: *E. necator* mycelium growth was slowed down from 1 to 2 dpi and restricted, sporulation was delayed to 7 dpi and was limited to 2–3 conidia per conidiophore at 10–11 dpi. Susceptible genotypes did not influence the mycelium growth, sporulation appeared at 4–5 dpi, reached 5–6 conidia per conidiophore and produced, on average, 2 times more conidia at 10–11 dpi (Additional file 8: Table S4; Additional file 9: Fig. S5). The resistance observed in the Caucasian accessions was not as effective as the genotypes carrying either *Run1, Ren5* or *Ren6*, which halt the pathogen hyphal growth and sporulation, therefore offering total resistance [29, 37, 38]. The partial resistance observed in this study had similar effects to those observed in genotypes carrying the *Ren1* and *Ren7* [18, 37, 39] and, finally, resulted in a significantly lower severity of the disease and capability of *E. necator* to establish new infections.

In our populations the resistance segregation was neither qualitative as suggested for *Run1*, *Ren4* and *Ren6* loci [16, 21, 37] nor quantitative as observedon *V. rupestris* [40]. The Mendelian segregation (Table 1) suggests the presence of a major genetic factor for the trait under observation; while the occurrence of more continuous resistance degrees (Fig. 1) would suggest the presence of further complementary determinants.

### A major QTL controls the resistance to *E. necator* in Caucasian grapevines

The GBS approach [41] performed well in our study despite the challenges represented by high grape heterozygosity, which might generate erroneous SNP calling, high-percentages of missing data and heterozygote under-calling [40, 42]. The two parental maps, each of about 1250 cM and 2400 markers, divided in 19 LG (Fig. 2; Table 2; Additional file 4: Fig. S3a and b) had a marker order consistent with the ‘PN40024’ grape reference genome 12X.v2 (Additional file 5: Fig. S4) [43] and agreed with previous GBS-derived linkage maps on the length of linkage groups and marker density (e.g. [40, 44, 45]). However, ‘Glera’ chr 13 displayed distorted segregations for all markers (Fig. 3). Islands of markers with distorted segregations may be common in interspecific crosses (e.g. [44, 46]), but they were also observed in crosses between *V. vinifera* cultivars (e.g. [47]). Distorted segregations may be unpredictable and occur because of post-zygotic lethal combinations that influence the viability of zygotes, germination of seeds and seedlings survival [44]. In our case an haplotypic region on the lower part of chr 13 of ‘Glera’ was defective and not inherited in the progeny. These pieces of evidence may suggest the presence of a new locus responsible for the gamete selection in *V. vinifera* in addition to the ones described by Riaz et al. [46] on the chr 14. Markers with distorted segregations can determine spurious linkage, erroneous marker order and imprecise QTL analysis [48]. However, maintaining only SNP not affecting the marker order and distances compared to ‘PN40024’ genome sequence allowed the genetic map of ‘Glera’ chr 13 to be completed. The segregation distortion described did not affect the detection of the resistance QTL because it interested only the markers of ‘Glera’ the susceptible cross parent of the mapping population.

All approaches adopted in the QTL analysis (interval mapping and multiple QTL research) identified a single major locus for resistance to *E. necator* on chr 13 of ‘Shavtsitska’ (Fig. 4; Table 3; Additional file 6: Table S2). The phenotyping data were all performant in mapping the QTL: the LOD peaks scores were different depending on variable, experiment and dpi considered but they were always significant and located the QTL in the same interval of 2.2 cM at 47 cM on the LG (Fig. 4; Table 3; Additional file 6: Table S2). The rAUDPC indexes, which summarized the infection progress for pathogen mycelium growth and sporulation, resulted as being the most informative data and explained up to 80% of the phenotypic variance (Table 3). In QTL mapping, the methods of phenotypic data collection, which comprise standardized sampling, handling, infection processing and rating, are as important as the genetic design and analysis. Our results were constant and reproducible and evidenced the effectiveness of the phenotyping strategy in describing the phenotypic patterns as well as the genetic of the trait studied. The properties describing the locus identified in ‘Shavtsitska’ (Table 3), which were further confirmed by investigations on the ‘Tskhvedianis tetra’ cross population (Fig. 6), showed that the QTL is a promising source of resistance to *E. necator*. It will possibly be important to test the Caucasian resistance simultaneously with various pathogen isolates and experimental conditions (e.g. environments) to understand and confirm the solidity and performance of the identified QTL. According to Ramming et al. [49], race-specific resistances were identified in *Vitis* spp. from the native area of *E. necator* in North America where coevolution between the pathogen and the host has been take place for a long time [50]. However, this possibility is more difficult to verify in Europe because of the bottleneck of *E. necator* introduction and the resulting low genetic pathogen diversity which exists in the continent [50].

There are currently 14 known QTL associated with resistance to *E. necator* (7; www.vivc.de) and the locus of ‘Shavtsitska’ was positioned in chr 13 between 16.8 and 18.2 Mb on ‘PN40024’(Fig. 5). Overlap the ‘Shavtsitska’ QTL, in the interval between 11.3 and 18.4 Mb, Hoffman et al. [18] identified the locus *Ren1* that was mapped starting from the ‘Kishmish vatkana’ SSR-based genetic map. Subsequently, the genetic region was further saturated with SSR markers and *Ren1* was delimited to the same area of 1.4 Mb of ‘Shavtsitska’ resistance locus. In our study, we report the first high-density genetic map of a *V. vinifera* variety resistant to *E. necator* and demonstrate the power of the GBS approaches for QTL mapping and quickly narrowing a region of interest [42].

We showed that the resistance to *E. necator* of Caucasian grapevines is coded by a major and effective gene. On the contrary, resistance to *P. viticola* in Caucasian germplasm appeared to be controlled by three different minor loci [8]. Both the introduction and pyramiding of major, in particular, and minor loci are important to define promising and durable resistance traits. Our results therefore strongly increase the interest in Caucasian grape accessions for breeding resistant grape cultivars [51]. Caucasian varieties carrying resistance to both *P. viticola* and *E. necator* may be the most valuable germplasm. However, the cross-checking of the results of our paper and that of Sargolzaei et al. [8] did not show Caucasian accessions carrying the resistance loci to both pathogens, but an analysis at a larger scale needs to be carried out.

### Origin of the resistance to *E. necator* in the Caucasian grapevines

The screening of ‘Shavtsitska’ and ‘Tskhvedianis tetra’ populations with the SC8-0071-014 and Sc47_20 SSR markers [19], located in ‘PN40024’ chr 13 at 16.87 and 18.24 Mb, respectively, revealed that the allele 149 of SC8-0071-014 and allele 208 of Sc47_20 are in coupling with the Caucasian resistance to *E. necator*. We extended the SSR analysis to 103 Caucasian grapes preserved at the CREA-VE germplasm repository, discovering that the haplotype 149–208 was shared by twenty-five varieties (Table 4; Additional file 7: Table S3). These results suggest that resistance to powdery mildew could be very frequent in Caucasian grape germplasm. We could not phenotypically characterize the Caucasian grapevines but the literature reported eleven of those varieties as partially resistant to *E. necator* [28]. For six of those grape accessions our molecular analysis showed the presence of the Caucasian resistant haplotype in chr 13 (Additional file 7: Table S3). Other five phenotypically resistant accessions did not share the same haplotype and this would suggest a more complex genetic landscape behind the resistant trait. However, only an extended phenotypic survey in the same experimental conditions, together with the collected molecular data, could provide clearer insights into the spread of resistance to *E. necator* in the Caucasian cultivated germplasm. Currently, our genetic findings are consistent with the Riaz et al. [52] study, which identified in the same genomic region of chr 13 of a Caucasian *V. vinifera* subsp. *sylvestris* a resistance QTL to *E. necator*.

Our research, in addition to other studies, identified the resistance to *E. necator* in many *V. vinifera* grapevines of different geographic areas (Caucasus and Central Asia) and collected evidence that its inheritance is shared by wild and cultivated *V. vinifera* subspecies [18, 19, 24, 52]. This information and the long history of grapes isolation in the Caucasus region [53, 54] suggests that the resistance trait might have been inherited from a *V. vinifera* progenitor thousands of years ago and conserved in Caucasian cultivars until today. In the ancestor/s, probably, the region evolved to fight different fungi-caused diseases, conserving an array of R-genes over time [19]. Maintenance of the trait in *V. vinifera* through domestication and until today was probably not intentional. The literature supports the hypothesis that *E. necator* co-evolved in North America on native wild *Vitis* spp. [50] and does not report powdery mildew disease in Europe and Asia before the nineteenth century [55]. It is less likely that the resistance developed recently in the Caucasian germplasm because the historic time of co-evolution between local grapevines and the pathogen has been too brief. The resistance haplotype does not appear to result from an interspecific introgression into ‘Shavtsitska’ and ‘Tskhvedianis tetra’, possibly through a chance cross with an American grapevine introduced in the area, because the Caucasian accessions showed purely *V. vinifera* genomes in resequencing studies (Magris G., Di Gaspero G., Morgante M. pers. comm.). However, we cannot exclude that either natural or intentional selection took place in the region last two centuries [56, 57], when the pressure of *E. necator* on grapevine cultivation became evident. Such a selection could explain the high frequency of resistance haplotype 149–208 within the Caucasian *V. vinifera*.

Anyway, Gur et al. [58] recently identified an *E. necator* strain in Israel genetically differentiated from those characterized in Europe and North America, proposing a non-American origin for it and possibly an Asian one. This hypothesis could explain the presence of resistance to *E. necator* in *V. vinifera*, and also in other Asian *Vitis* spp. [18, 19, 24, 52], with the co-evolution theory. However, the Gur et al. [58] suggestion is in contrast to common notions on *E. necator* origin and centres of differentiation (e.g. [50, 55]) and a more in-depth study would be necessary to confirm their new proposals.

### Genetic basis of resistance to *E. necator* in Caucasian and Central Asia grape germplasm

The mapping of co-located QTL for the resistance to *E. necator* in many and unrelated *V. vinifera* revealed a high complexity of the investigated region in chr 13, that encompasses some megabase from upstream to downstream of the mapped loci [19]. This would suggest a question: are the Caucasian and Central Asian resistant *V. vinifera* grapevines, which carry different marker haplotypes, sharing the same resistance genes or are we dealing with different resistance determinants developed starting from a common ancestor?

Phenotypic information collected in our research often showed distinct responses to *E. necator* of Caucasian grapevines (in particular ‘Shavtsitska’) and ‘Kishmish vatkana’. However, the phenotypic resistance of the Caucasian and Central Asian grape accessions, due to the trait variations associated to the loci [24, 39, 52], does not allow to clearly confirm whether the genetic basis of resistances is different or not.

We analysed the region of the QTL mapped in ‘Shavtsitska’ in the‘PN40024’ grape reference genome 12X.v2 [43] and found a single putative disease resistance gene, namely RPP13-like protein 1, an NBS-LRR type R protein with a putative amino-terminal leucine zipper (Additional file 10: Table S5). However, approximately one Mb upstream of the QTL, there are six RPP13-like protein 1 (5 + the one of the QTL) and four At3g14460, a gene isolated first in *A. thaliana* that also belongs to the class of NB-LRR microorganism defence response genes. It is interesting to note that the RPP13-like protein of the QTL contains multiple splicing variants. According to several authors, RPP13 is prone to undergoing evolutionary amino acid divergence within the LRR domain, which might create alleles deputed to recognise different strains of a pathogen [59]. These pieces of evidence would suggest investigating in the future the candidate region in ‘Shavtsitska’, a task that was not possible to accomplish in this study due to the low coverage of the genome in the produced reads.

The literature would suggest other cases where regions rich in R-genes encompass multiple resistance loci. For instance, *Ren4*, from *V. romanetii* [21], and *Run2*, from *V. rotundifolia* [60], loci map in the same position of chr 18 of ‘PN40024’; furthermore, *Run2* is associated to two resistant haplotypes (*Run2.1* and *Run2.2*) that originate from close *V. rotundifolia* accessions [60]. The *Ren1* region in chr 13 contains numerous genes encoding NBS-LRR proteins and appears prone to producing genetic variation [19]. The natural selection and evolution mechanisms at the basis of R-genes clusters [13, 19, 61–63] could also have developed Caucasian and Central Asian *V. vinifera* accessions with different resistance genes and/or unique combinations of resistance factors. We therefore consider that the identified resistance locus in ‘Shavtsitska’, contains a member of a cluster of R-genes, of which the region is rich, and to name such a variant as *Ren1.2* because it is linked with, or possibly allelic to, the previously described *Ren1*.

Information collected until now does not allow us to concluded whether grapevines from Central Asia and the Caucasus share the same resistance genes or not. Further narrowing of the genetic region of chromosome 13 explored up to now, as well as comparative sequence analysis and deep transcriptomic study would allow to focus on the precise genetic differences. More precise phenotyping and histochemical observations could complement the information on the origin of the resistance variation and on the mechanism behind the trait.

## Conclusions

The mapping study on the two grape varieties ‘Shavtsitska’ and ‘Tskhvedianis tetra’ (*V. vinifera* subsp. *vinifera*) native to the Caucasus revealed the possible presence of a new locus of resistance to *Erysiphe necator* that mapped in chromosome 13, near the region where *Ren1* locus of Central Asian grapevines is located. The genomic region surrounding *Ren1*, in the grape ‘PN40024’ grape reference genome, resulted as being very rich in NBS-LRR resistance genes and prone to produce genetic variations. The Caucasian resistant accessions have an allelic profile different from the *Ren1*-carrying genotypes from Central Asia. We speculated that Eurasian *V. vinifera* grapes could have developed multiple and independent resistance genes located on chromosome 13 around *Ren1* genetic region.

‘Shavtsitska’, ‘Tskhvedianis tetra’ and their resistant progeny are characterized by a partial resistance to *E. necator* able to delay and limit the growth and sporulation of the pathogen and the severity of its disease in laboratory conditions. As a result of the extended genetic screening into Caucasian grapevines, the resistance trait appears to be widely diffused in such germplasm. Caucasian accessions might therefore be interesting for grape breeding programmes because they are cultivated varieties with a *V. vinifera* genetic background and pleasant agronomic characteristics. The new investigated source of resistance to *E. necator* can be introduced in breeding lines in one or limited cross generations, in the perspective of producing new elite cultivars with pyramided resistance loci for a more sustainable viticulture.

## Methods

### Plant material

Representatives grapevine accessions native of Caucasus region were collected in the frame of the project COST FA1003 Action “East - West Collaboration for Grapevine Diversity and Exploration and Mobilization of Adaptive Traits for Breeding” and were maintained in the grape germplasm repository of ERSA-Agenzia Regionale per lo Sviluppo Rurale, Centro Pilota per la Viticoltura (ERSA) in Gorizia province (Friuli-Venezia Giulia region, Italy) until 2010 [53]. A total of 105 Caucasian *V. vinifera* subsp. *vinifera* from ERSA have been reproduced and grown at the CREA - Research Centre for Viticulture and Enology (CREA-VE) grape germplasm collection in Treviso province (Veneto region, Italy; 45°51′07.6″N 12°15′28.6″E) since 2010. The Additional File 7: Table S3 provides a detailed list of the 105 Caucasian accessions analysed in this study and knowledge on their true-to-type genetic profile (Migliaro pers. comm.; https://www.vivc.de/). Interinstitutional agreements permitted to collect the plant samples from the germplasm repositories and to use the plant accessions to produce and collect the seeds used in this research.

In 2018, to obtain seeds to generate mapping populations we performed several grape control crosses at CREA-VE germplasm collection: the Caucasian accessions ‘Shavtsitska’ and ‘Tskhvedianis tetra’, for which we had precise information on their genetic origin, resistance degree (Magris G., Di Gaspero G., Morgante M. pers. comm [28]) and availability of in field plants*,* were cross pollinated with the two susceptible *V. vinifera* varieties ‘Glera’ and ‘Chardonnay’. In 2019, we sowed the seeds of the designed cross population 50041 - ‘Chardonnay’ x ‘Tskhvedianis tetra’ and 50042 - ‘Shavtsitska’ x ‘Glera’ at INRAE-Centre Grand Est-Colmar UMR 1131 SVQV (INRAE-SVQV) (Colmar, France). The offspring was genetically verified by means of molecular markers and 270 true-to-type progenies of each population were grown in two-litre pots in a mixture of sand-perlite-lapilli. Replicates of the cross parents and several control grapevine genotypes (characterized by different degrees of resistance to *E. necator* and/or carrying specific resistance loci), among which the varieties ‘RV1–22–8-78’ (carrying *Run1*), ‘Kishmish vatkana’ (carrying *Ren1*), ‘Johanniter’ (carrying *Ren3* and *Ren9*) and ‘Cabernet sauvignon’ (carrying no locus), were produced at INRAE-SVQV from green cuttings and maintained in a greenhouse together with the progeny for the subsequent phenotypic comparative evaluations. The plants were grown at 28 °C with 16 h light and 8 h dark photoperiod. Shoots were periodically pruned to limit the vegetation and guarantee the presence of young apical leaves for the phenotyping bioassays. Pests and diseases were managed by sprayings every 2 weeks.

### Disease evaluation

The phenotypic resistance of parental plants and offspring was studied by using leaf discs bioassays managed as described in Calonnec et al. [64], with some modifications, and evaluating the powdery mildew infection features during the pathogen life cycle [65].

An isolate of *E. necator* was obtained at INRAE in Colmar from *V. vinifera* plants. The isolate was maintained and multiplied in vitro on leaves of ‘Cabernet sauvignon’. For this purpose, every 10 days some young and shiny leaves (3–8 cm in diameter) were: disinfected by an incubation for 4 min in a 50 g/l sodium hypochlorite solution, rinsed in three consecutive baths of sterile water for 4 min each, dried between sterile paper towels, placed on a medium containing 10 g/l agar and 0.015 g/l natamycin in Petri dishes with the adaxial surface up and petioles partially trimmed, and finally inoculated by blowing *E. necator* conidia from ten-day-old infected leaves through a custom-made settling tower.

For the phenotyping bioassays, young, shiny and expanded leaves of 2–4 cm in diameter from the shoot apex (from the second to fourth position) of each plant with an optimal growth were collected and treated as described in the paragraph above. The sample discs were then excised with a cork borer and placed in Petri dishes on a wet filter paper disc lying on the agar medium and inoculated with 600–800 conidia/cm^2^ of *E. necator* optimally grown for ten-day on leaves of ‘Cabernet sauvignon’.

Inoculated Petri dishes were incubated in a climatic chamber at 23 °C with a photoperiod of 16 h light and 8 h dark.

The plants response and pathogen development at 1-2-3 days post-infection (dpi) were investigated through a histochemical and scanning-electron-microscope (SEM) study from June 2019 to July 2020. In the histochemical bioassays, the two cross parents ‘Shavtsitska’ and ‘Glera’, seventeen of their offspring and the control genotypes were studied. For each individual, three leaf discs of 1 cm in diameter were observed at 1, 2, 3 dpi in two replicated experiments. The fungal structures were stained with Trypan-Blue as described in Agurto et al. [30] and Vogel & Somerville [66] with minor modifications. Leaf discs were cleared by washing three times for 30 min with a solution of ethanol-96% and acetic acid-100% (3:1 by volume), stored in lactoglycerol (glycerol 99.5%, lactic acid 90% and water 1:1:1 by volume) for 12 h at room temperature, stained with a Trypan-Blue water solution (0.01% weight/volume) for 15 min and finally stored in lactoglycerol. Discs were mounted on slides for bright-field microscopy visualization by Zeiss Axio Imager M2 (Zeiss, Oberkochen, Germany) with 100x magnifications. One-hundred germinated conidia per disc were categorised in 4 classes according to their development:

- Class 0-conidia showing only the appressoria;
- Class 1-conidia showing the primary hypha;
- Class 2-conidia showing the primary and the secondary hypha;
- Class 3-conidia showing three hyphae and/or branched hyphae.

Observations with SEM were made with a Hitachi TM-1000 microscope (Hitachi, Tokyo, Japan).

In new experiments, *E. necator* infections were extensively evaluated in cross population 50041 at 2-4-7-9-11 dpi and for population 50042 at 3-5-7-10 dpi. A total of 67 plants of population 50041 (58 were evaluated twice and 9 once) and 264 plants of population 50042 (58 were evaluated twice and 9 once) were studied. In each replicate one disc in 2 cm of diameter per progeny and up to four discs per parental and control plant were observed. At each dpi, four areas on the leaf discs were scored for the following infection variables: pathogen mycelium growth, sporulation intensity, mean number of conidia per conidiophore and presence-absence of plant necrosis. Pathogen mycelium and sporulation were scored with two independent scales with five classes each, according to OIV-455 scale [67] with some modifications:

- 9-absence of pathogen structures in the area;
- 7-presence of few short hyphae/few conidiophores;
- 5-mycelium/conidiophores sparse with low density or spread in colonies;
- 3-dense mycelium/conidiophores on most of the leaf disc area;
- 1-dense mycelium/conidiophores covered all the observed area.

Discs were observed under a Zeiss Stemi 508 stereomicroscope (Zeiss, Oberkochen, Germany) at 64x magnification. After the last evaluation, discs of plants of population 50042 were stored in 1.5 ml tubes at − 20 °C. Subsequently conidia were suspended in 300 ul of Tween-20 water solution (0.05% volume/volume) and counted with Malassez counting chamber. Conidia counts were square root transformed (RQSP) before data analysis. The relative Area Under Disease Pressure Curve (rAUDPC) [68] was calculated for *E. necator* mycelium growth and sporulation intensity with the averaged scores per disc per dpi: AUDPC values were calculated by the simple midpoint (trapezoidal) rule, then they were divided with the maximum possible AUDPC for the experiment to obtain the rAUDPC.

### DNA extraction and genotyping

For each germinated seedling and cross parent, total DNA was extracted from a single young expanded leaf (about 50 mg of tissue). Samples were collected in 96-well plates, maintained for 1 min in liquid nitrogen, ground to a fine powder by a Tissue-Lyser II instrument (Qiagen, Hilden, Germany) (30 Hz for 45 s twice) and treated with DNeasy 96 Plants DNA kits (Qiagen, Hilden, Germany). Modifications were made to the manufacturer’s protocol to improve DNA yield and quality as follows: PVP-30 (1.5% weight/volume) was added to the lysis buffer (AP1) prior to heating and elution was performed with 80 ul of buffer (AE) heated at 65 °C.

The SSR markers VVMD5 [69], VVMD27, VVMD28 [70], VrZag79 [71] and VMCNG4b9 (*Vitis* Microsatellite Consortium - Agrogene, Moissy Crameyel, France) were used to screen the cross populations 50041 and 50042 for contaminants. PCR reactions were performed following the conditions described in Blasi et al. [72]. The SSR SC8-0071-014 and Sc47_20 [19] were screened in a subsample of individuals of both breeding populations and in the 105 Caucasian varieties conserved at CREA-VE (Additional file 7: Table S3) as described in De Nardi et al. [73]. PCR fragments were analysed with GeneMapper 4.0 software (Thermo Fisher Scientific, Waltham, Massachusetts, USA).

DNA of ‘Shavtsitska’, ‘Glera’ and 184 progenies (population 50042) were quantified with Qubit 3.0 (Thermo Fisher Scientific, Waltham, Massachusetts, USA) and verified by gel electrophoresis at 1% agarose medium EEO with GelRed 1:10000 (Biotium, Fremont, California, USA). The 184 progenies were chosen on the basis of the ‘quality’ of phenotyping data: the 159 individuals phenotyped three times and 25 individuals phenotyped twice and showing stable phenotypes in the experiments (rAUDPC variation for pathogen sporulation < 0.08) were preferentially retained. About 1500–3000 ng DNA was dried at 65 °C for 2 h and delivered to ‘The Elshire Group’ (Palmerston North, New Zealand) for the libraries preparation, GBS analysis and SNP calling.

The GBS data were generated following the Elshire et al. [41] method with the following modifications: 100 ng of genomic DNA and 3.6 ng of total adapters were used; the genomic DNAs were restricted with *ApeKI* enzyme and the libraries were amplified with 18 PCR cycles. The libraries were sequenced by Illumina HiSeq X (Illumina, San Diego, California, USA) that generated 150 bp paired end reads. The demultiplexing based on combinatorial barcoding was performed with Kevin Murray’s axe-demux v.0.3.3 [74]. Sequencing data have been deposited in the Sequence Read Archive (SRA) of NCBI and are available at the following link: https://www.ncbi.nlm.nih.gov/bioproject/PRJNA725652/. The reads for both ends of the pair-end data were combined into individual per-sample files and aligned to the *V. vinifera* ‘PN40024’ grape reference genome 12X.v2 [43] using Bowtie2 v.2.4.1 [75]. The alignments were subsequently analysed with Stacks v.2.5 [76] and the Kinship using GBS with Depth adjustment program (KGD) v.0.7.0 [77] to output the final SNP dataset in a .vcf file.

### Genetic mapping and QTL analysis

The SNP dataset was preliminarily analysed with Perl [78] scripts described in Hyma et al. [45]: probable genotyping errors were corrected based on the genotype quality (by using a threshold of 20) and SNP having a genotyping rate above 90%, an error rate lower than 5% and two segregating alleles were retained. ‘Shavtsitska’ and ‘Glera’ genotypes were extracted in R software environment [79] with the vcfr package v. 1.11.0 [80] functions. SNP showing homozygous, heterozygous and missing data in both parents were discarded. SNP with conflicting genotypes in the duplicate parental samples were also discarded. Then, markers were divided into the putative belonging chromosomes according to their position on ‘PN40024’ and in two datasets (Additional file 11: Table S6a and Table S6b) according to the parent from which they segregated in order to build the two parental linkage maps following the pseudo-testcross mapping strategy [81]. Markers with significantly distorted segregations (*p*-values for chi-square tests < 0.001) and co-locating were eliminated from the dataset. The SNP association within chromosomes was verified with the Minimum Spanning Tree (MSTmap) algorithm [82] (mstmap function in ASmap package v. 1.0.4 [83] with default parameters) and either SNP separated from the belonging LG and in weak linkage (low mean association-LOD value/high recombination fraction) with the neighbouring markers were eliminated. Individuals with a number of crossover/double crossover far from that usually observed in the population (between 10 and 50) were manually discovered and eliminated. Probable genotyping errors were corrected to missing data based on genotypes logarithm of odds (LOD) scores (LOD > 3). The final marker order was defined with the MSTmap algorithm [82], while the final marker distances were calculated with the Lander-Green algorithm (est.map function in qtl package v. 1.46.2 [84];) by the Kosambi mapping function [85]. Chromosome numbers and their orientation were defined according to the ‘PN40024’ genome sequence [43].

Genotypic and phenotypic data of 183 individuals of population 50042 were utilized together to conduct the QTL analysis by using the software R (qtl package v. 1.46.2 [84, 86]). Data collected at 3-5-7-10 dpi for pathogen mycelium development, sporulation intensity and presence-absence of plant necrosis were investigated first. After that, conidia counts obtained by Malassez chamber and the rAUDPC indexes were also explored. Individual and averaged experiments data were all analysed. Interval Mapping (IM) was performed by using the Expectation–Maximization (EM) algorithm and non-parametric models with the Kruskall-Wallis test were verified when phenotypic data residuals were on the edge of normal distribution. The search for more independent and/or interacting resistance loci was refined following the QTL model selection approach [86] and Multiple-QTL-Mapping method [87]. Genome wide LOD significance thresholds per each phenotype were calculated by permutation tests (n.perm = 1000 and *p*-values< 0.05) [86]. Bayes credible intervals were determined for the significant identified QTL (α = 0.95) [86]. Resistance QTL identified in the cross parents were projected onto the ‘PN40024’ genome sequence to extract the informative recombinants for the loci.

QTL segregation in the cross population 50041 was verified by testing the phenotypic differences between the SSR SC8-0071-014 and Sc47_20 haplotype-derived groups: T-tests (*p*-value< 0.05) and linear models for data of rAUDPC for pathogen mycelium growth and sporulation were calculated by software R (t.test and lm functions in stat package [79]).

## Supplementary Information

**Additional file 1: Table S1.** Descriptive statistics for the phenotypic data recorded for the Caucasian cross populations during *E. necator* infections.

**Additional file 2: Figure S1.** Distributions of the phenotypic data related to *E. necator* resistance of the cross populations.

**Additional file 3: Figure S2.** Pairwise correlations between phenotypic data recorded for the cross populations during the *E. necator* infections.

**Additional file 4: Figure S3.** Parental genetic maps.

**Additional file 5: Figure S4.** Genetic maps marker order and distances compared to marker physical position on grape reference genome.

**Additional file 6: Table S2.** Results for the interval mapping for the resistance to *E. necator* of ‘Shavtsitska’.

**Additional file 7: Table S3.** Screening with SSR of the Caucasian germplasm conserved at CREA-VE.

**Additional file 8: Table S4.** Phenotypic data of the resistance to *E. necator* recorded in different bioassays for the Caucasian cross parents and their progenies.

**Additional file 9: Figure S5** Representative phenotypes at the stereomicroscope of seedlings resistant and susceptible to *E. necator*.

**Additional file 10: Table S5.** Resistance genes predicted in the QTL of resistance to *E. necator* in chr 13 in *V. vinifera*.

**Additional file 11: Table S6.** SNP dataset analysed in R-software for the construction of the parental maps.

## Data Availability

The phenotypic and genetic data generated and analysed during this study are included in this published article and its supplementary information files. The sequencing data are available from Sequence Read Archive (SRA) database of NCBI (BioProject ID: PRJNA725652; https://www.ncbi.nlm.nih.gov/bioproject/PRJNA725652/).

## Abbreviations

BAM: Binary alignment map
bp: Bays pair
chr: Chromosome
cM: Centi-Morgans
dpi: Days post-infection
EM: Expectation–maximization
GBS: Genotyping by sequencing
HR: Hypersensitive response
IM: Interval mapping
KGD: Kinship using GBS with depth adjustment program
LG: Linkage group
LOD: Logarithm of odds
Mb: Megabase
MST: Minimum spanning tree
OIV: Organisation Internationale de la Vigne et du Vin
PCD: Programmed cell death
PCR: Polymerase chain reaction
QTL: Quantitative trait loci
R (−genes): Resistance
rAUDPC: Relative area under disease pressure curve
ROS: Reactive oxygen species
*Ren*: Resistance to *Erysiphe necator*
*Rpv*: Resistance to *Plasmopara viticola*
*Run*: Resistance to *Uncinula necator*
SEM: Scanning electron microscope
SNP: Single nucleotide polymorphism
SSR: Single sequence repeat
vcf: Variant call format
