# Supplementary material for: Construction of a high-density genetic map and detection of a major QTL of resistance to powdery mildew (Erysiphe necator Sch.) in Caucasian grapes (Vitis vinifera L.)
Source: BMC Plant Biol. 2021 Nov 11;21:528. doi: 10.1186/s12870-021-03174-4 (PMC8582213; doi:10.1186/s12870-021-03174-4)
Supplement: Supplementary file 2 — Additional file 2: Figure S1. Distributions of the phenotypic data related to E. necator resistance of the cross populations. [file 12870_2021_3174_MOESM2_ESM.docx]

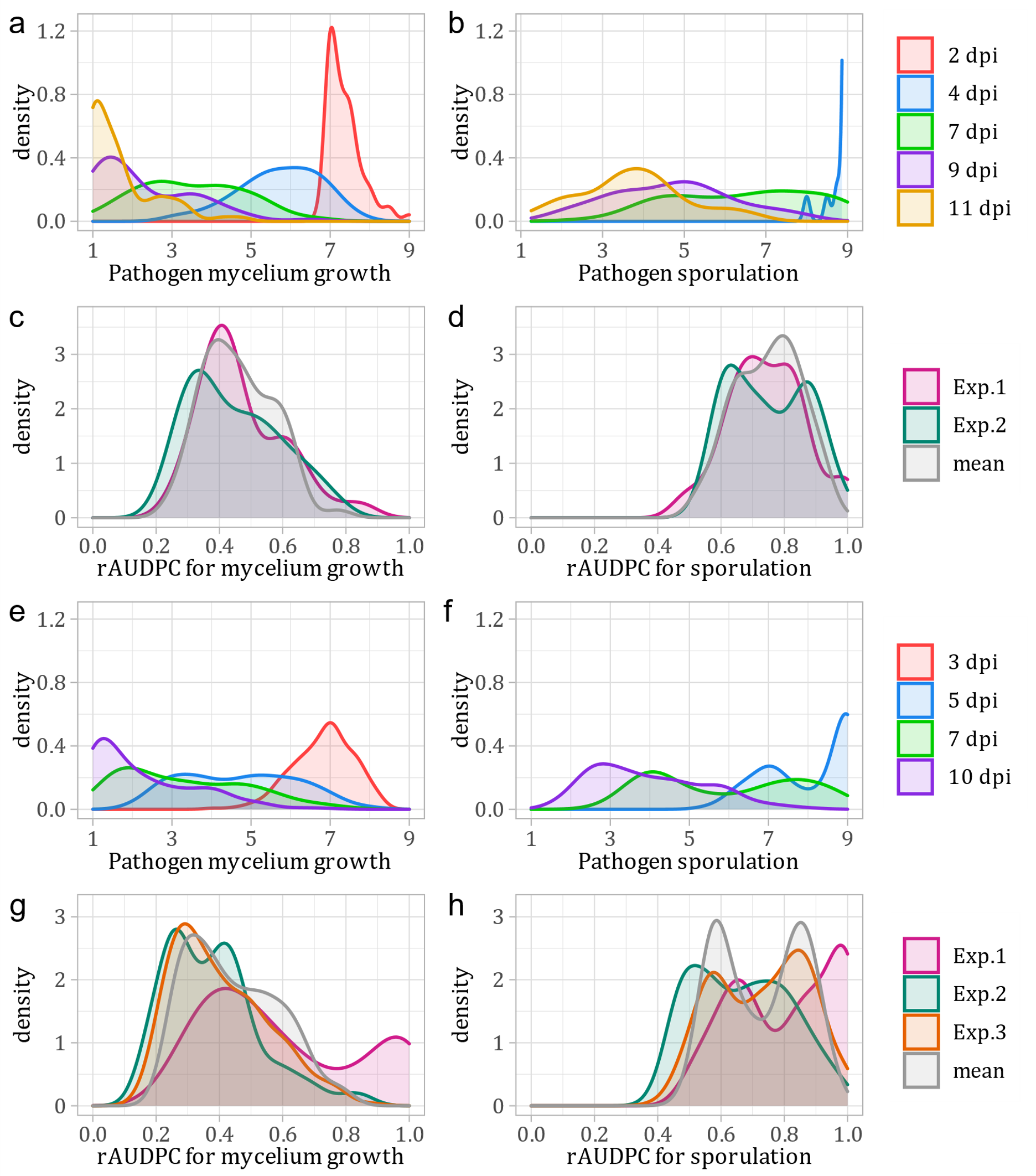
**Figure S1.** The plots show the distributions of some phenotypic data collected in the evaluation of the resistance to *E. necator* of the cross populations 50041-‘Chardonnay’ x ‘Tskhvedianis tetra’ (plot a-b-c-d) and 50042-‘Shavtsitska’ x ‘Glera’ (plot e-f-g-h). Plots a-b-e-f display the distributions of the averaged values per offspring (calculated from 2 or 3 experiments replicates) for *E. necator* mycelium growth and sporulation intensity scored with two independent scales developed according to OIV-455 descriptors (x-axis) recorded in different dpi (different colours). Plots c-d-g-h display the distributions of the calculate rAUDPC values per offspring (x-axis) for *E. necator* mycelium growth and sporulation intensity in the different experiment replicates (different colours). The y-axis represents the probability density (probability per unit on the x-axis), the actual probabilities can be calculated through the area under the curve (the total area integrates to one).
