# Supplementary material for: Construction of a high-density genetic map and detection of a major QTL of resistance to powdery mildew (Erysiphe necator Sch.) in Caucasian grapes (Vitis vinifera L.)
Source: BMC Plant Biol. 2021 Nov 11;21:528. doi: 10.1186/s12870-021-03174-4 (PMC8582213; doi:10.1186/s12870-021-03174-4)
Supplement: Supplementary file 3 — Additional file 3: Figure S2. Pairwise correlations between phenotypic data recorded for the cross populations during the E. necator infections. [file 12870_2021_3174_MOESM3_ESM.docx]

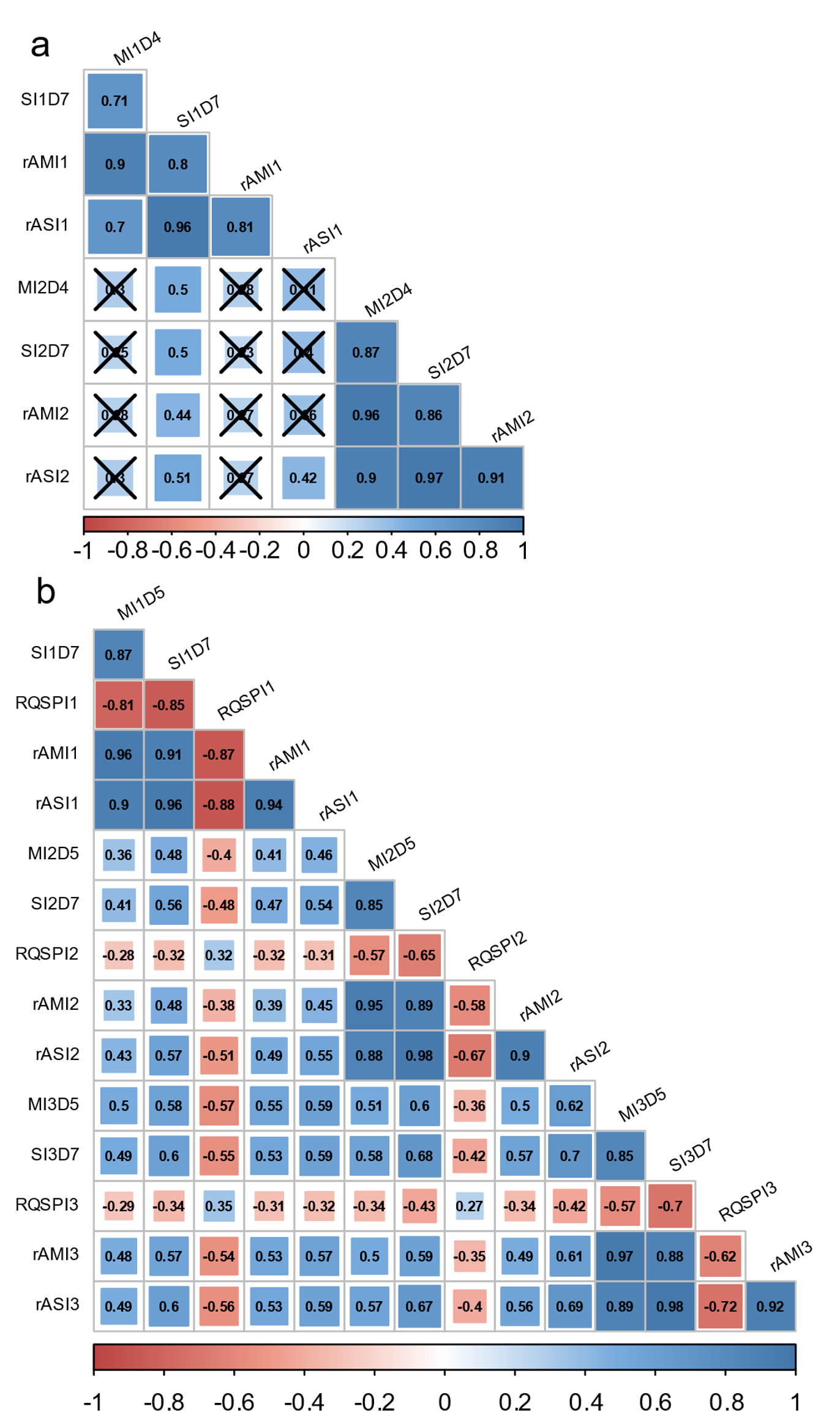
**Figure S2.** Pairwise correlations (Spearman’s rank correlation) between phenotypic data recorded for the breeding populations 50041-‘Chardonnay’ x ‘Tskhvedianis tetra’ (plot a) and 50042-‘Shavtsitska’ x ‘Glera’ (plot b) during the *E. necator* infections on leaf discs. The acronyms designate the phenotypic data series: the first letter specifies the infection variable (M→mycelium growth, S→sporulation intensity, RQSP→conidia counts by Malassez counting chamber square-root transformed, rAM→rAUDPC for mycelium growth and rAS→rAUDPC for sporulation intensity), the second letter-number specifies the experiments replicate (I1-I2-I3→inoculation/replicate 1, 2 or 3) and the last letter-number specifies the dpi of observation (D5-D7→dpi 5 or7). Spearman’s rank correlation coefficients crossed represent correlation with p-value > 0.001.
