# Supplementary material for: Construction of a high-density genetic map and detection of a major QTL of resistance to powdery mildew (Erysiphe necator Sch.) in Caucasian grapes (Vitis vinifera L.)
Source: BMC Plant Biol. 2021 Nov 11;21:528. doi: 10.1186/s12870-021-03174-4 (PMC8582213; doi:10.1186/s12870-021-03174-4)
Supplement: Supplementary file 4 — Additional file 4: Figure S3. Parental genetic maps. [file 12870_2021_3174_MOESM4_ESM.pdf]

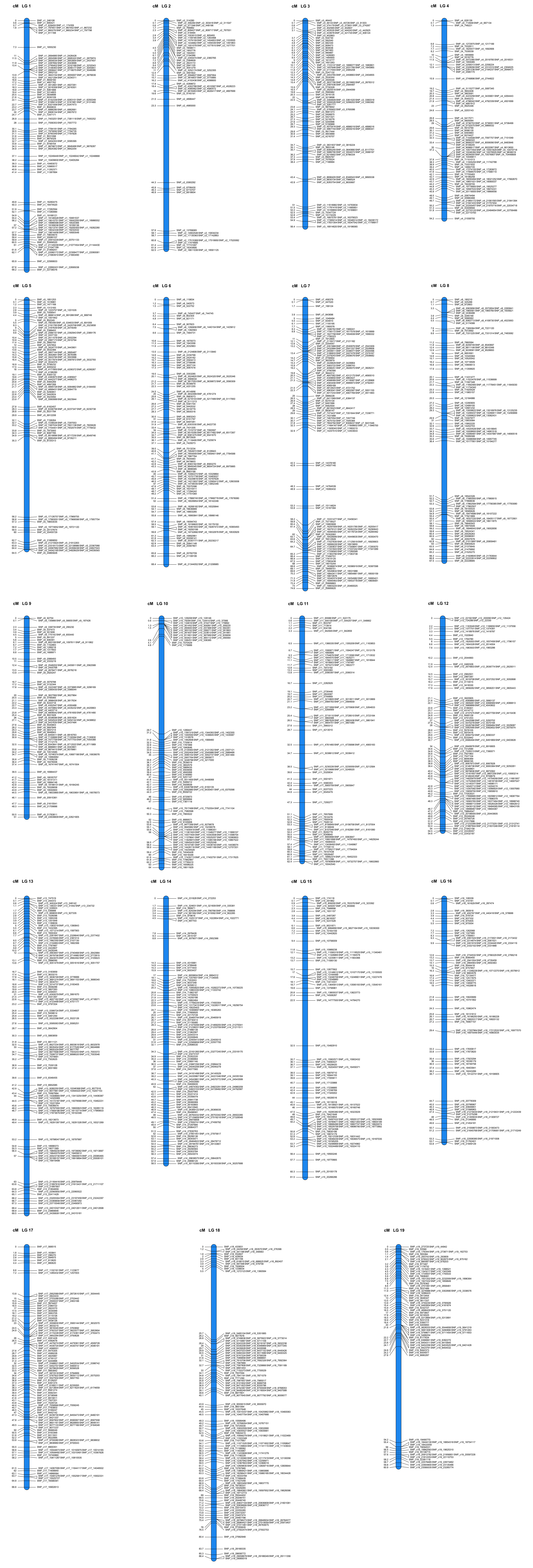

Figure S3a 'Shavtsitska' genetic map. Genetic distances are calculated by Kosambi mapping function and expressed in 'centi Morgan' (cM) on the left of each linkage group (LG). LG numbers and SNP positions are defined according to grape reference genome 'PN40024' 12X.v2 (Canaguier et al. 2017).

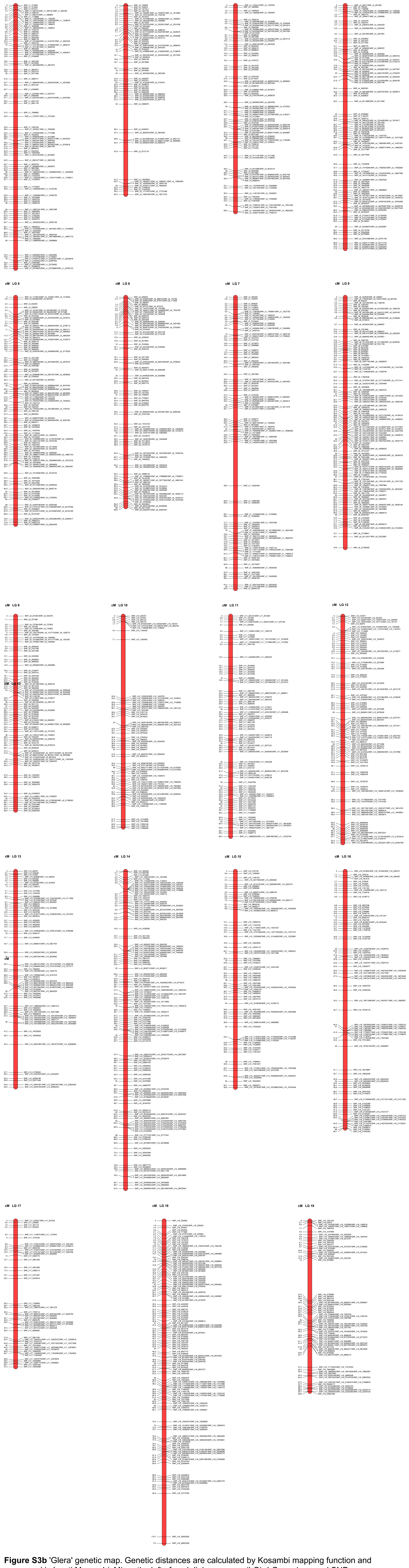

Figure S3b 'Glera' genetic map. Genetic distances are calculated by Kosambi mapping function and expressed in 'centi Morgan' (cM) on the left of each linkage group (LG). LG numbers and SNP positions are defined according to grape reference genome 'PN40024' 12X.v2 (Canaguier et al. 2017).
