# Supplementary material for: Construction of a high-density genetic map and detection of a major QTL of resistance to powdery mildew (Erysiphe necator Sch.) in Caucasian grapes (Vitis vinifera L.)
Source: BMC Plant Biol. 2021 Nov 11;21:528. doi: 10.1186/s12870-021-03174-4 (PMC8582213; doi:10.1186/s12870-021-03174-4)
Supplement: Supplementary file 5 — Additional file 5: Figure S4. Genetic maps marker order and distances compared to marker physical position on grape reference genome. [file 12870_2021_3174_MOESM5_ESM.docx]

**
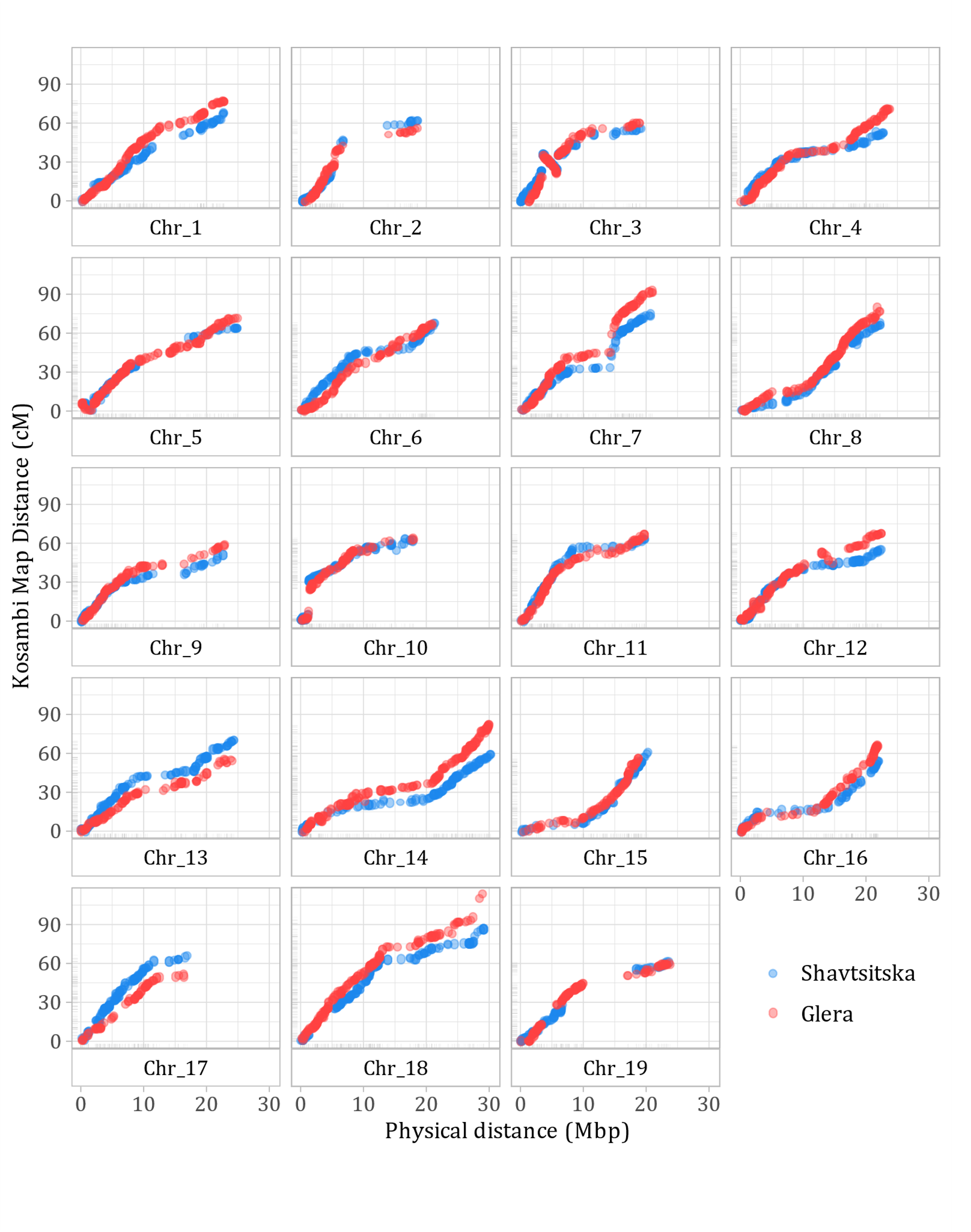
Figure S4.** Dot plot for SNP markers utilized for the development of the parental linkage maps of ‘Shavtsitska’ (blue) and ‘Glera’ (red). On the x-axis the SNP physical distances on grape reference genome ‘PN40024’ 12X.v2 (Canaguier et al. 2017) while on y-axis the SNP genetic distances.
