# Supplementary material for: Construction of a high-density genetic map and detection of a major QTL of resistance to powdery mildew (Erysiphe necator Sch.) in Caucasian grapes (Vitis vinifera L.)
Source: BMC Plant Biol. 2021 Nov 11;21:528. doi: 10.1186/s12870-021-03174-4 (PMC8582213; doi:10.1186/s12870-021-03174-4)
Supplement: Supplementary file 9 — Additional file 9: Figure S5 Representative phenotypes at the stereomicroscope of seedlings resistant and susceptible to E. necator. [file 12870_2021_3174_MOESM9_ESM.docx]

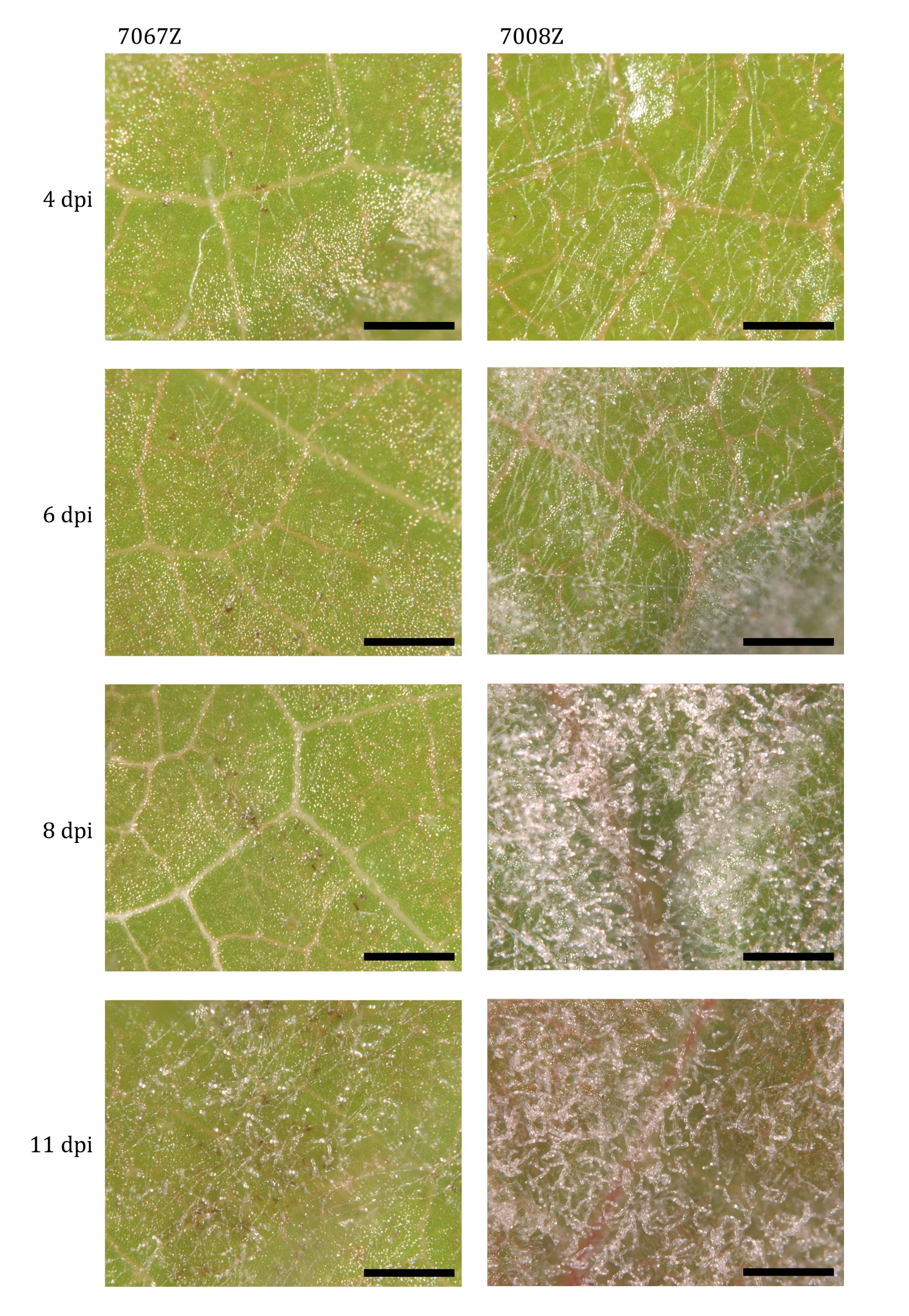


**Figure S5***. Erysiphe necator* development on 7067Z (resistant) and 7008Z (susceptible) seedling discs (50042 – ‘Shavtsitska’ x ‘Glera’ population) at 4-6-8-11 dpi. On the resistant seedling the pathogen mycelium growth and sporulation intensity are delayed and more limited in comparison to the susceptible seedling. Furthermore, on 7067Z the number of conidia per conidiophores is usually lower and the plant necrotic response is more frequent. Magnification x64. Scale bar 500 μm.
